# Contrast-enhanced endoscopic ultrasound and fine-needle biopsy of a rare mediastinal mass: a mediastinal schwannoma

**DOI:** 10.1055/a-2163-1544

**Published:** 2023-09-21

**Authors:** Dario Ligresti, Dario Quintini, Ilaria Tarantino, Alessandro Bertani, Agita Jukna, Giacomo Emanuele Maria Rizzo, Mario Traina

**Affiliations:** 1 Endoscopy Service, Department of Diagnostic and Therapeutic Services, IRCCS – ISMETT, Palermo, Italy; 2 Department of Surgical Oncological and Gastroenterology Sciences, University of Padua, Padua, Italy; 3 Unit of Thoracic Surgery and Lung Transplantation, Department for the Treatment and Study of Cardiothoracic Diseases and Cardiothoracic Transplantation, IRCCS – ISMETT, Palermo, Italy; 4 Pathology Unit, Department of Diagnostic and Therapeutic Services, IRCCS – ISMETT, Palermo, Italy; 5 Department of Surgical, Oncological and Oral Sciences, University of Palermo, Palermo, Italy

Contrast-enhanced endoscopic ultrasonography-guided fine-needle biopsy (CE-EUS-FNB) is an important, minimally invasive tool for the diagnosis of mediastinal masses. The specimen obtained allows a wider range of analyses to be performed and a better description of morphology and immunophenotype compared with fine-needle aspiration (FNA). The main advantages of CE-EUS include real-time imaging of microvascularity and microperfusion, and impressively good detail resolution 1 .

We present the case of a 20-year-old woman with Horner syndrome. On computed tomography scan, a nodular mass of approximately 7 cm in diameter in the left apical pleura was described, with compression of both the pulmonary parenchyma and subclavian artery ( Fig. 1 ). After multidisciplinary board discussion, it was decided that tissue sampling should be performed for histological definition ( Video 1 ).

**Fig. 1:**
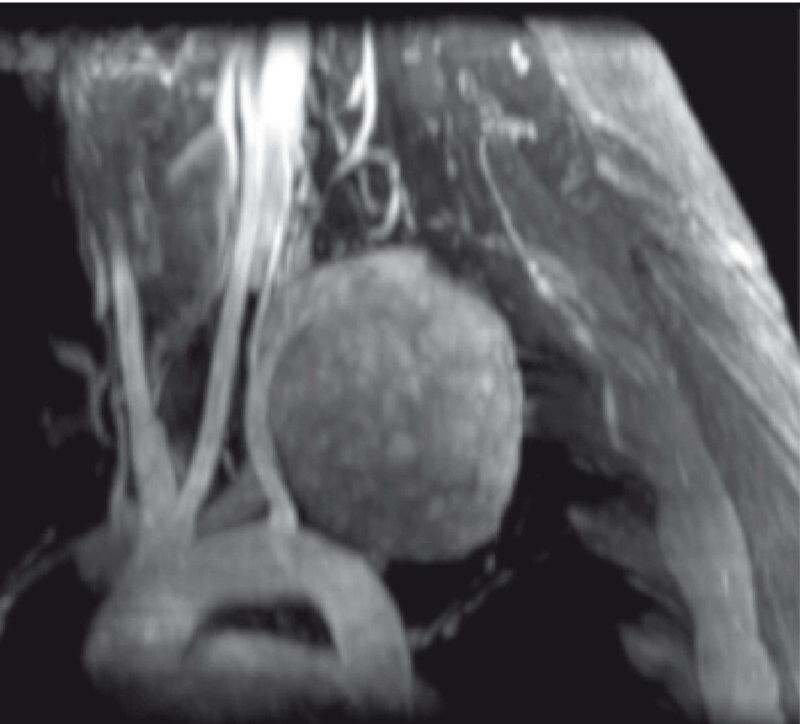
Computed tomography 3 D reconstruction with a nodular mass of approximately 7 cm in diameter in the left apical pleura with compression of the subclavian artery.

**Video 1** Contrast-enhanced endoscopic ultrasonography examination and fine-needle biopsy of a mediastinal schwannoma.


We performed CE-EUS, which revealed a 7-cm hypoechoic mass in close apposition to the aortic arch, left subclavian artery, and left common carotid artery ( Fig. 2 ), with diffuse and inhomogeneous hypo-enhancement following contrast injection ( Fig. 3 ) (Sonovue; Bracco, Milan, Italy). A transesophageal EUS-FNB was performed with a 22-G needle (SharkCore; Medtronic, Minneapolis, Minnesota, USA). Our pathologists defined the tumor as a benign peripheral nerve sheath tumor, most compatible with schwannoma, thanks to the features described by the wide range of immunohistochemical stains (positivity for S100, GFAP, SOX10, D2.40, and negativity for CAM5.2, CD117, MelanA, CD34) ( Fig. 4 ). Thus, the patient underwent a thoracoscopic mass resection, with a regular postoperative course. The surgical specimen ( Fig. 5 ) confirmed the final diagnosis of schwannoma.

**Fig. 2:**
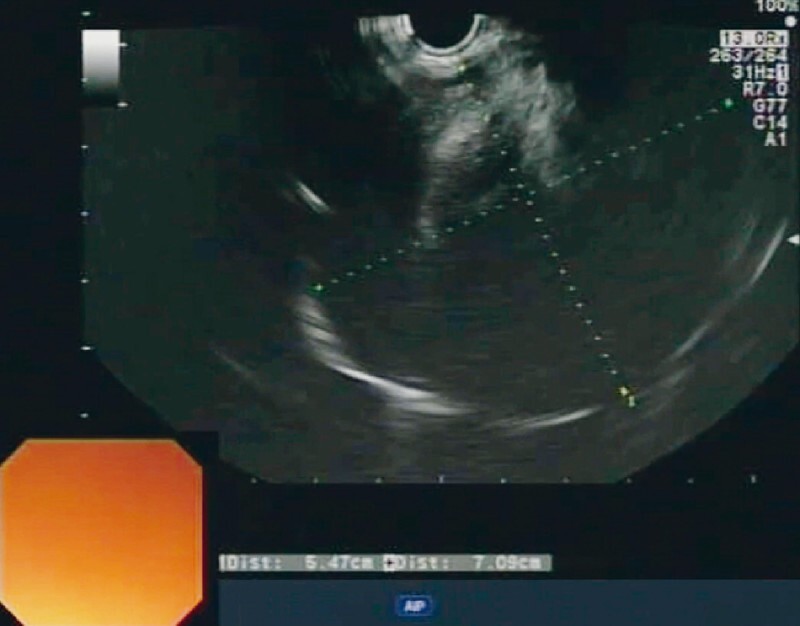
Endoscopic ultrasound view of the lesion showing a 7-cm hypoechoic mass.

**Fig. 3:**
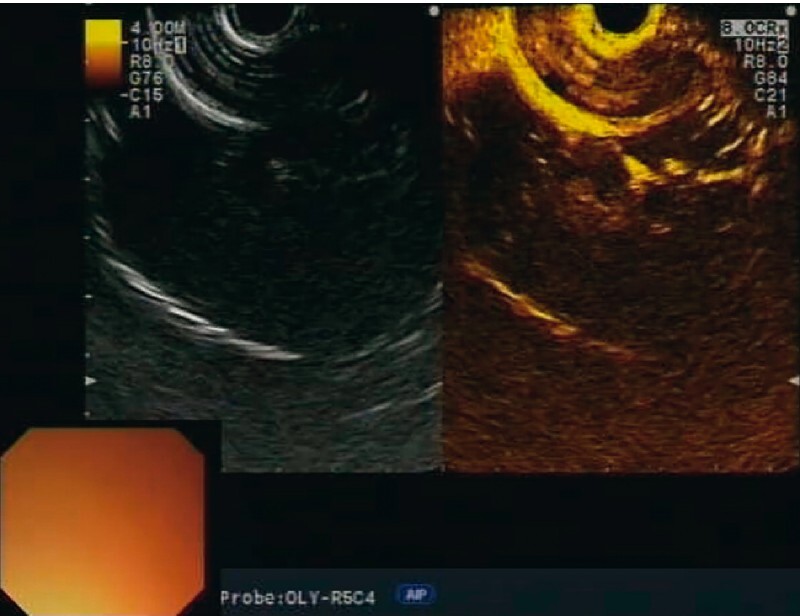
Endoscopic ultrasound view of the lesion showing a diffuse and inhomogeneous hypo-enhancing pattern following contrast injection.

**Fig. 4:**
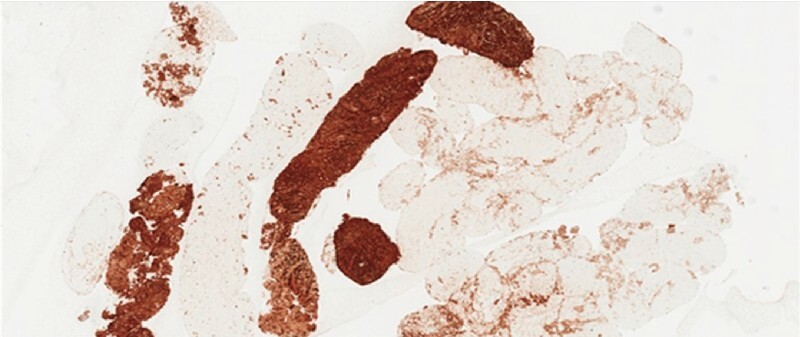
Tumor cells demonstrated strong and diffuse expression of S100 (original magnification × 3).

**Fig. 5:**
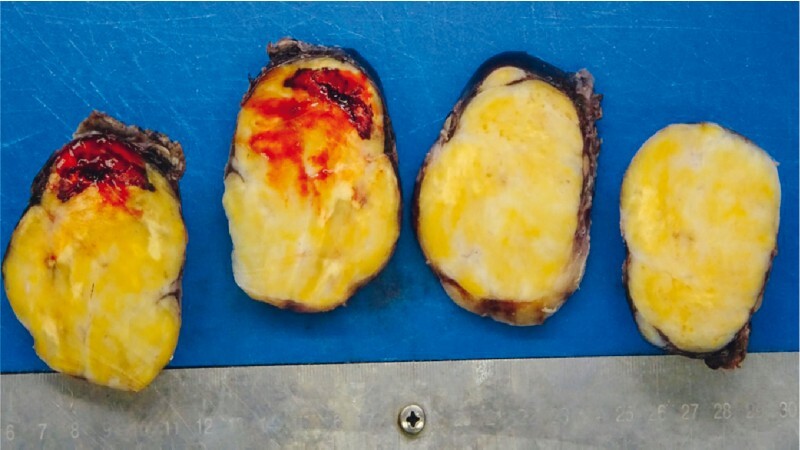
Macroscopic appearance of the resected tumor in sections, with smooth surface and firm, light-tan tissue with focal patches of hemorrhage.

Mediastinal schwannoma is a rare mediastinal mass 2 , and only a single case of EUS-FNA cytological diagnosis has been reported 3 . To the best of our knowledge, neither contrast-enhancement behavior nor endoscopic FNB has been described for this rare mediastinal lesion. Nevertheless, the histological specimen acquired by EUS-FNB allows for the use of a wider range of immunohistochemical stains, increasing the specificity of diagnosis. Furthermore, contrast-enhanced evaluation offers real-time guidance for EUS-FNB and is likely to increase sensitivity.

Endoscopy_UCTN_Code_CCL_1AF_2AC

## Notes

Endoscopy E-Videos

https://eref.thieme.de/e-videos
*E-Videos* is an open access online section of the journal *Endoscopy* , reporting on interesting cases and new techniques in gastroenterological endoscopy. All papers include a high-quality video and are published with a Creative Commons CC-BY license. Endoscopy E-Videos qualify for HINARI discounts and waivers and eligibility is automatically checked during the submission process. We grant 100% waivers to articles whose corresponding authors are based in Group A countries and 50% waivers to those who are based in Group B countries as classified by Research4Life (see: https://www.research4life.org/access/eligibility/ ). This section has its own submission website at https://mc.manuscriptcentral.com/e-videos
